# Decreasing Birth Weight Is Associated with Adverse Metabolic Profile and Lower Stature in Childhood and Adolescence

**DOI:** 10.1371/journal.pone.0119433

**Published:** 2015-03-11

**Authors:** José G. B. Derraik, Deborah L. Rowe, Wayne S. Cutfield, Paul L. Hofman

**Affiliations:** 1 Liggins Institute, University of Auckland, Auckland, New Zealand; 2 School of Nursing, Faculty of Medical and Health Sciences, University of Auckland, Auckland, New Zealand; Children's National Medical Center, Washington, UNITED STATES

## Abstract

**Objective:**

We aimed to evaluate the association of birth weight SDS with insulin resistance, blood pressure, and auxology in children and adolescents born 23–42 weeks of gestation.

**Methods:**

We studied 143 singleton children and adolescents aged 9.3 ± 3.3 years (range 2.0–17.9 years). Clinical assessments included insulin resistance measured by HOMA2-IR, auxology, and blood pressure from sphygmomanometer measurements. Continuous associations were examined, and stratified analyses carried out. For the latter, participants were divided into those of below-average birth weight (BABW, <0 SDS) and above-average birth weight (AABW, ≥0 SDS).

**Results:**

Irrespective of gestational age, lower birth weight SDS was associated with progressively greater HOMA2-IR (p<0.0001) and higher fasting insulin concentrations (p<0.0001). Decreasing birth weight SDS was associated with higher systolic (p = 0.011) and diastolic (p = 0.006) blood pressure. Lower birth weight SDS was also associated with decreasing stature (p<0.010). The BABW group was ~40% more insulin resistant than AABW participants (p = 0.004), with the former also displaying fasting insulin concentrations 37% higher (p = 0.004). BABW participants were 0.54 SDS shorter than those of higher birth weight (p = 0.002). On average, BABW participants had not met their genetic potential, tending to be shorter than their parents (p = 0.065). As a result, when corrected for parents' heights, BABW participants were 0.62 SDS shorter than those born of higher birth weight (p = 0.001). Sub-group analyses on participants born appropriate-for-gestational-age (n = 128) showed that associations of birth weight SDS with both insulin resistance and stature remained (although attenuated).

**Conclusion:**

Decreasing birth weight SDS (even within the normal range) is associated with adverse metabolic profile and lower stature in children and adolescents.

## INTRODUCTION

Numerous studies have shown that low birth weight is associated with short- and long-term adverse health outcomes [1–4]. Children and adolescents born small-for-gestational-age have higher blood pressure [5] and lower insulin sensitivity [6,7], as well as experiencing modest deficits in cognition, learning, attention, and academic achievement [8,9]. In adulthood, studies have shown low birth weight to be associated with higher blood pressure [2,4,5], lower insulin sensitivity and increased incidence of type 2 diabetes mellitus [10,11], greater cardiovascular disease risk [1], and increased mortality [3].

However, most studies have examined associations with birth weight expressed in kilograms or grams. These are less accurate measures of health at birth as they do not take into account the gestational age of the individual. For example, among those born at term, a 3.0 kg boy born at 37 weeks of gestation has a birth weight in the 50^th^ centile, while the same weight at 41 weeks would place him in the 5^th^ centile.

Thus, studies using standard deviation score (SDS) rather than kilograms provide a more accurate assessment of birth weight effects, as it accounts for gestational age. As a result, we aimed to assess the association of birth weight SDS with insulin resistance, blood pressure, and auxology in children and adolescents born across the gestational age range.

## METHODS

### Ethics

Ethics approval was provided by the Northern Ethics Committee (Ministry of Health, New Zealand). Written informed consent was obtained from parents or guardians. Verbal and written informed consent was also obtained from participants, except for the youngest children unable to write who provided verbal consent in the presence of parents or guardians. This study was performed in accordance with all appropriate institutional and international guidelines and regulations for medical research, in line with the principles of the Declaration of Helsinki.

### Participants

Children and adolescents were recruited for a study examining the effects of prematurity on endocrine regulation of growth [12]. All participants were of New Zealand European ethnicity, naturally conceived, born from singleton pregnancies, and with normal neurological development. Participants were excluded if they had chronic illness, chromosomal or syndromal diagnosis, or were receiving medication known to affect growth.

### Assessments

Clinical assessments were carried out during a single visit to the Maurice and Agnes Paykel Clinical Research Unit (Liggins Institute, University of Auckland), after an overnight fast. Heights and weights were measured on parents and participants, with body mass index (BMI) subsequently calculated. Each child's birth weight, height, and BMI were transformed into SDS [13,14]. Mid-parental height SDS (MPHSDS) was calculated [15], and the child’s height SDS was individually corrected for their genetic potential (target height) using the formula: "height SDS—MPHSDS". Total body fat percentage was assessed by bioelectrical impedance analysis (Tanita TBF-105, Tanita Corporation, Tokyo, Japan).

Venous blood samples were collected and frozen at -80°C for later analysis. Plasma glucose was measured using an automated random-access analyser (Hitachi, Tokyo, Japan) with inter-assay coefficient of variation 1.2%. Insulin levels were determined by enzyme immunoassay (Abbott Laboratories, Chicago, Illinois, USA) with inter-assay coefficient of variation <5%.

Homeostasis model assessment of insulin resistance (HOMA2-IR) was obtained from fasting insulin and glucose values using the HOMA2 calculator v2.2.3 (www.dtu.ox.ac.uk/homacalculator/). Blood pressure was measured at the clinic by the same researcher using a standard mercury sphygmomanometer, with an appropriately-sized cuff on the non-dominant arm. Measurements were recorded on each child while seated and after a 5-minute rest. All subjects had pubertal development assessed by the same experienced paediatric endocrinologist. Participants were defined as pubertal by having Tanner stage 2 breast development in girls and testicular volume >3 ml in boys or evidence of adrenarche.

### Statistical analyses

Continuous associations with birth weight SDS were assessed using simple linear correlations and linear mixed regression models, with the latter including family identification number as a random factor to account for the clustering of siblings. Important confounding factors were controlled for in the analyses, including gestational age, pubertal status, age, and sex. Other factors were included as required, depending on the parameter of interest: for outcomes associated with glucose homeostasis—BMI SDS; for body composition—maternal BMI; and for blood pressure—height (instead of age) and BMI SDS. Stratified analyses were also carried out comparing participants of below-average birth weight (BABW, <0 SDS) versus those of above-average birth weight (AABW, ≥0 SDS) using linear mixed regression models as described above. Baseline comparisons between the two groups were carried out using one-way ANOVA and Fisher's exact tests. Note that the interaction between birth weight SDS and sex was assessed for all outcomes. In addition, sub-group analyses were carried out separately for prepubertal and pubertal participants, as well as only on those participants who were born appropriate-for-gestational-age (birth weight >-2 and <2 SDS).

Univariate analyses were carried out in Minitab v.16 (Pennsylvania State University, State College, PA, USA), while multivariate analyses were performed in SAS v.9.3 (SAS Institute Inc., Cary, NC, USA). All statistical tests were two-tailed and maintained at a 5% significance level. Demographic data are presented as means ± standard deviation (SD). Outcome data from stratified analyses are presented as model-adjusted means (estimated marginal means adjusted for the confounding factors in the models), with associated 95% confidence intervals.

## RESULTS

**Fig 1:**
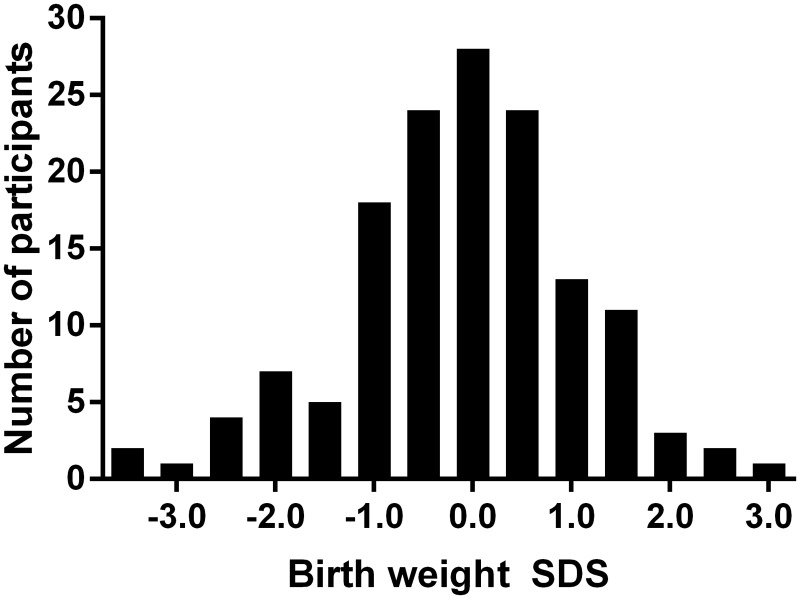
Histogram showing the frequency distribution of children studied according to birth weight standard deviation scores (SDS).

We studied 143 children and adolescents aged 9.3 ± 3.3 years (range 2.0–17.9 years), including 69 males (48%). Participants had a mean birth weight of-0.11 ± 1.17 SDS (range-3.39–2.90 SDS) spread over a Gaussian distribution (Fig. 1). Sixty (42%) participants were born preterm (<37 weeks of gestation), 11 (8%) born small-for-gestational-age (SGA, birth weight ≤-2.0 SDS), and 4 (3%) born large-for-gestational-age (birth weight ≥2 SDS). Gestational age of participants ranged from 23 to 42 weeks of gestation.

### Continuous associations

Decreasing birth weight SDS was correlated with increasing HOMA2-IR (r = -0.30; p<0.0001), fasting insulin concentrations (r = -0.30; p<0.0001), and diastolic blood pressure (r = -0.22; p = 0.008). Lower birth weight SDS was also correlated with lower height SDS (r = 0.22; p = 0.009) and lower height SDS–MPHSDS (r = 0.18; p = 0.032).

**Fig 2:**
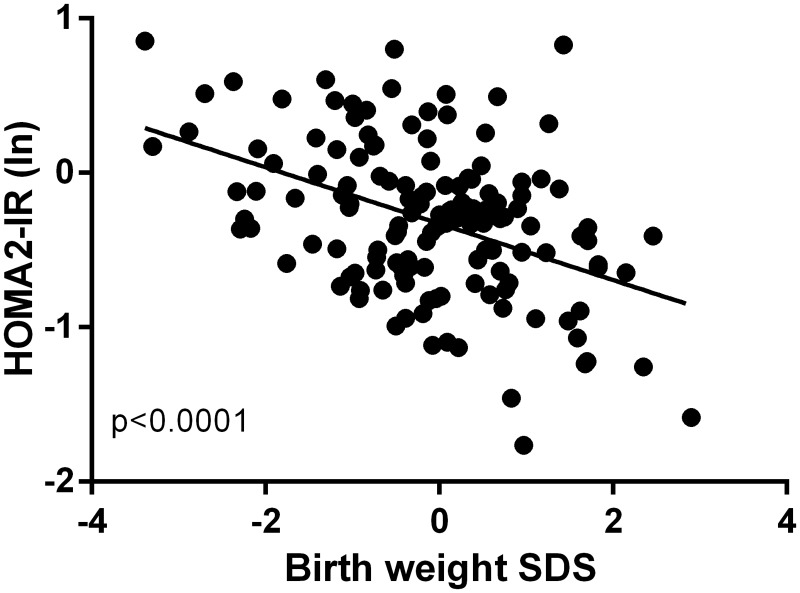
The association between birth weight standard deviation scores (SDS) and HOMA2-IR (expressed as the natural logarithm). Individual data points have been adjusted for confounding factors (including gestational age, pubertal status, and sex) in the multivariate models.

**Fig 3:**
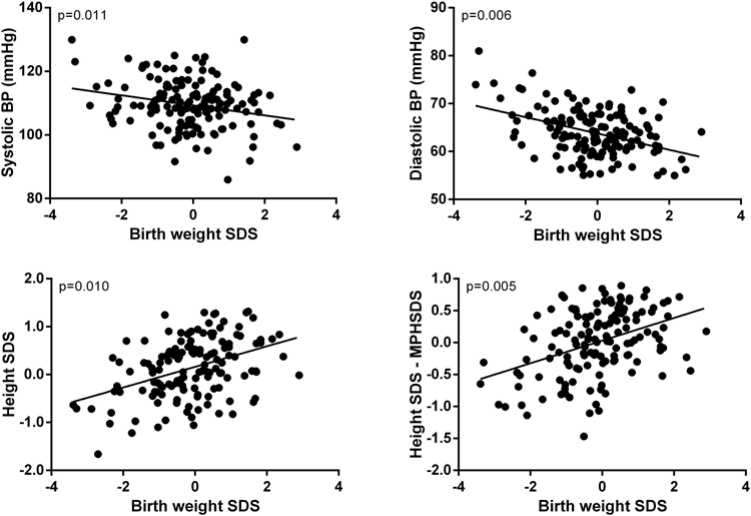
The association of birth weight SDS with blood pressure (BP) and stature. Individual data points have been adjusted for confounding factors (including gestational age, pubertal status, and sex) in the multivariate models.

Multivariate models corroborated the above findings. Lower birth weight SDS was associated with progressively greater HOMA2-IR (p<0.0001) and higher fasting insulin concentrations (p<0.0001) (Fig. 2). Decreasing birth weight SDS was associated with higher systolic (β = -2.27; p = 0.011) and diastolic (β = -1.86; p = 0.006) blood pressure (Fig. 3). Lower birth weight SDS was also associated with decreasing height SDS (β = 0.21; p = 0.006) (Fig. 3). Importantly, lower birth weight SDS was associated with decreasing stature adjusted for parents' heights (height SDS–MPHSDS; β = 0.22; p = 0.010) (Fig. 3).

Sub-group analyses on the 93 prepubertal children showed that decreasing birth weight SDS was strongly associated with higher HOMA2-IR (β = -0.16; p = 0.0004) and fasting insulin concentrations (β = -1.34; p = 0.0003), and tended to be associated with higher systolic (β = -1.71; p = 0.064) and diastolic (β = -1.41; p = 0.053) blood pressures. Among the 50 pubertal participants, lower birth weight was associated with decreasing height SDS (β = 0.49; p = 0.002), decreasing height SDS–MPHSDS (β = 0.52; p = 0.001), increasing diastolic blood pressure (β = -3.90; p = 0.005), and increasing pulse rate (β = -6.22; p = 0.003).

### Stratified analyses

BABW and AABW participants were of similar age and sex ratio, as well as having a similar proportion of participants in puberty or born preterm (Table 1). The BABW group was nearly 40% more insulin resistant than AABW participants, as measured by both HOMA2-IR (p = 0.004) and fasting insulin concentrations (p = 0.004; Table 1).

**Table 1:** Characteristics of the 143 children studied according to their birth weight standard deviation scores (SDS).

		Below-average birth weight (<0 SDS)	Above-average birth weight (≥0 SDS)	p-value
**n**		76	67	
**Demography**	Age (years)	9.3 ± 3.2	9.2 ± 3.4	0.70
	Sex ratio (males/females)	35/41	34/33	0.62
	Born preterm	46%	37%	0.31
	Pubertal status (in puberty)	36%	30%	0.48
**Auxology**	Height SDS	-0.10 (-0.35–0.14)	0.44 (0.17–0.71)	0.002
	Height SDS–MPHSDS	-0.26 (-0.53–0.02)	0.36 (0.06–0.66)	0.001
	BMI SDS	-0.08 (-0.34–0.18)	0.21 (-0.08–0.49)	0.11
	Total body fat (%)	19.3 (17.5–21.1)	19.7 (17.7–21.6)	0.76
**Glucose homeostasis**	HOMA2-IR	0.85 (0.72–1.00)	0.62 (0.52–0.74)	0.004
	Fasting glucose (mmol/l)	4.58 (4.48–4.68)	4.54 (4.44–4.65)	0.57
	Fasting insulin (mU/l)	6.71 (5.71–7.89)	4.90 (4.11–5.84)	0.004
**Cardiovascular parameters**	Systolic blood pressure (mmHg)	109.5 (106.5–112.5)	107.7 (104.4–111.0)	0.40
	Diastolic blood pressure (mmHg)	64.7 (62.4–67.0)	62.5 (59.9–65.0)	0.15
	Pulse rate (bpm)	81.3 (77.7–84.9)	79.4 (75.4–83.4)	0.44

Age data are mean ± SD; other data are means and 95% confidence intervals adjusted for confounding factors in the multivariate models.

BABW participants were 0.54 SDS shorter than those of higher birth weight (p = 0.002; Table 1). On average, BABW participants were not meeting their genetic potential, tending to be shorter than their parents (p = 0.065). As a result, when corrected for parents' heights, BABW participants were 0.62 SDS shorter than those born of higher birth weight (p = 0.001; Table 1). There were no observed differences in cardiovascular parameters between groups.

Note that there were no observed interactions between birth weight SDS and sex, indicating the absence of any sex-specific responses. Sub-group analyses examining prepubertal and pubertal participants separately yielded similar results for anthropometry in both groups (data not shown). However, pubertal BABW participants had pulse rate that was significantly higher than the AABW group (79.2 vs 67.4 bpm; p = 0.015).

### Appropriate-for-gestational-age

Sub-group analyses examining only participants born appropriate-for-gestational-age showed that associations of birth weight SDS with insulin resistance and height remained, although attenuated (Table 2). There were however, no observed associations with blood pressure.

**Table 2:** Characteristics of 128 children born appropriate-for-gestational-age according to their birth weight standard deviation scores (SDS) Age data are mean ± SD; other data are means and 95% confidence intervals adjusted for confounding factors in the multivariate models.

		Below-average birth weight (<0 SDS)	Above-average birth weight (≥0 SDS)	p-value
**n**		65	63	
**Demography**	Age (years)	9.2 ± 3.2	9.3 ± 3.5	0.97
	Sex ratio (males/females)	30/35	33/30	0.60
	Born preterm	42%	37%	0.59
	Pubertal status (in puberty)	32%	32%	0.99
**Auxology**	Height SDS	0.04 (-0.22–0.30)	0.47 (0.20–0.74)	0.013
	Height SDS–MPHSDS	-0.12 (-0.41–0.17)	0.41 (0.11–0.71)	0.006
	BMI SDS	-0.04 (-0.32–0.25)	0.27 (-0.03–0.56)	0.11
	Total body fat (%)	19.4 (17.4–21.4)	20.3 (18.3–22.3)	0.52
**Glucose homeostasis**	HOMA2-IR	0.82 (0.69–0.98)	0.65 (0.55–0.79)	0.052
	Fasting glucose (mmol/l)	4.58 (4.48–4.69)	4.54 (4.44–4.65)	0.60
	Fasting insulin (mU/l)	6.49 (5.44–7.73)	5.17 (4.31–6.20)	0.054
**Cardiovascular parameters**	Systolic blood pressure (mmHg)	107.1 (103.9–110.3)	107.5 (104.2–110.8)	0.87
	Diastolic blood pressure (mmHg)	63.1 (60.7–65.4)	62.0 (59.6–64.4)	0.49
	Pulse rate (bpm)	78.5 (74.5–82.4)	78.7 (74.6–82.7)	0.94

### Gestational age

Independently of birth weight, gestational age impacted on growth as previously reported [12]. Shorter gestational age was strongly associated with reduced height SDS (β = 0.074; p<0.0001) and failure to meet the genetic height potential (height SDS–MPHSDS; β = 0.064; p = 0.0005). Shorter gestation was also associated with lower BMI SDS (β = 0.058; p = 0.0008). However, gestational age was not associated with insulin sensitivity (as measured by HOMA2-IR or fasting insulin concentrations) or the cardiovascular parameters assessed.

## DISCUSSION

We showed that decreasing birth weight SDS was associated with an adverse metabolic profile (specifically reduced insulin sensitivity and higher blood pressure) as well as shorter stature in childhood and adolescence. While these findings were independent of gestational age, pubertal status, current adiposity, or parents' heights, decreasing gestational age was associated with progressively worse growth. Thus, the shortest children were those born both preterm and with low birth weight SDS. Importantly, subgroup analyses showed associations between birth weight SDS within the normal range and both insulin resistance and growth.

Individuals born of low birth weight or SGA have been shown to have reduced insulin sensitivity [6,7,16] and increased likelihood of developing type 2 diabetes [11,17]. Across the birth weight spectrum, a positive association with insulin sensitivity was observed in young adulthood [18]. Our study corroborates these findings, showing a marked increase in insulin resistance in those born with a birth weight below average (<0 SDS). Importantly, our data indicate that there appears to be no threshold beyond which insulin resistance starts to increase, but rather that it becomes progressively greater with decreasing birth weight SDS.

Low birth weight is associated with higher blood pressure in adolescence [19] and increased risk of developing hypertension in adulthood [17,20], with a systematic review concluding that birth weight is inversely related to blood pressure in childhood and adulthood [5]. Our observations are in agreement with the existing evidence, although findings were not supported by our stratified analyses. Our scatter plots and sub-group analyses indicate that, unlike the progressive change with insulin resistance, the increase in blood pressure seems to occur primarily among those born SGA (≤2 SDS).

One study has examined the final heights of individuals born of extremely low birth weight (500–999 g), and found that, although shorter, their stature was consistent with parents' heights [21]. In contrast, our participants of lower birth weight (<0 SDS) were yet to meet their genetic height potential, with another investigation in prepubertal subjects also observing that low-birth-weight children (<2,000 g) were shorter than their parents [22]. Although these findings may reflect the younger age of these subjects, at least one study has observed subnormal final height in SGA boys (but not SGA girls) [23]. Importantly, the observed association of birth weight SDS and growth were not due to effects of preterm birth [12], as gestational age was accounted for in all our analyses.

We did not observe any association between birth weight and BMI SDS or total body fat percentage. These findings are not particularly surprising, as the increased risk of overweight and obesity in low-birth-weight subjects occurs in later life and rarely before early adulthood. Increasing obesity has been reported in adults who were born of lower birth weight SDS [24] or preterm [25].

Although the data are conflicting, studies have shown an association between preterm birth and insulin sensitivity throughout the life course [26]. In this study, we have not observed an association between gestational age and insulin sensitivity, possibly due to the type of assessment used. In our research centre, indices of fasting insulin (e.g. HOMA2-IR) have been a poor indicator of peripheral insulin sensitivity, while stimulated measures (such as those obtained by an oral or intravenous glucose tolerance test) show more consistent abnormalities in preterm subjects [27,28]. Fasting insulin concentrations are considered to be a better reflection of hepatic insulin sensitivity, and thus the reduced insulin sensitivity observed in preterm children (with normal fasting insulin and abnormal intravenous glucose tolerance test) may reflect an isolated peripheral reduction in insulin sensitivity. In contrast, the elevated HOMA2-IR and fasting insulin concentrations we observed in children and adolescents with low birth weight SDS may reflect primarily a hepatic reduction in insulin sensitivity, which is consistent with findings in children born SGA [6].

The main limitation of our study was the wide age range of study subjects, including participants who were both pubertal and prepubertal. As puberty is known to be associated with increased insulin resistance [29] and changes in body composition [30], it was paramount that we controlled for pubertal status in our multivariate models examining the cohort as a whole.

In conclusion, decreasing birth weight SDS across the gestational age range is associated with adverse metabolic profile and shorter stature in children and adolescents. In particular, there was a progressive reduction in insulin sensitivity with decreasing birth weight SDS across the normal range.

## References

[1] Rich-Edwards JW, Stampfer MJ, Manson JE, Rosner B, Hankinson SE, Colditz GA, et al (1997) Birth weight and risk of cardiovascular disease in a cohort of women followed up since 1976. BMJ 315: 396–400. 9277603 10.1136/bmj.315.7105.396 PMC2127275

[2] Moore VM, Cockington RA, Ryan P, Robinson JS (1999) The relationship between birth weight and blood pressure amplifies from childhood to adulthood. J Hypertens 17: 883–888. 10419060 10.1097/00004872-199917070-00003

[3] Risnes KR, Vatten LJ, Baker JL, Jameson K, Sovio U, Kajantie E, et al (2011) Birthweight and mortality in adulthood: a systematic review and meta-analysis. Int J Epidemiol 40: 647–661. 10.1093/ije/dyq267 21324938

[4] Whincup P, Cook D, Papacosta O, Walker M (1995) Birth weight and blood pressure: cross sectional and longitudinal relations in childhood. BMJ 311: 773–776. 7580437 10.1136/bmj.311.7008.773 PMC2550786

[5] Law CM, Shiell AW (1996) Is blood pressure inversely related to birth weight? The strength of evidence from a systematic review of the literature. J Hypertens 14: 935–941. 8884547

[6] Hofman PL, Cutfield WS, Robinson EM, Bergman RN, Menon RK, Gluckman PD (1997) Insulin resistance in short children with intrauterine growth retardation. J Clin Endocrinol Metab 82: 402–406. 9024226 10.1210/jcem.82.2.3752

[7] Chiavaroli V, Giannini C, D'Adamo E, de Giorgis T, Chiarelli F, Mohn A (2009) Insulin resistance and oxidative stress in children born small and large for gestational age. Pediatrics 124: 695–702. 10.1542/peds.2008-3056 19651586

[8] Strauss RS (2000) Adult functional outcome of those born small for gestational age: twenty-six-year follow-up of the 1970 British Birth Cohort. JAMA 283: 625–632. 10665702 10.1001/jama.283.5.625

[9] O’Keeffe MJ, O’Callaghan M, Williams GM, Najman JM, Bor W (2003) Learning, cognitive, and attentional problems in adolescents born small for gestational age. Pediatrics 112: 301–307. 12897278 10.1542/peds.112.2.301

[10] Newsome CA, Shiell AW, Fall CHD, Phillips DIW, Shier R, Law CM (2003) Is birth weight related to later glucose and insulin metabolism?—a systematic review. Diabet Med 20: 339–348. 12752481 10.1046/j.1464-5491.2003.00871.x

[11] Harder T, Rodekamp E, Schellong K, Dudenhausen JW, Plagemann A (2007) Birth weight and subsequent risk of type 2 diabetes: a meta-analysis. Am J Epidemiol 165: 849–857. 17215379 10.1093/aje/kwk071

[12] Rowe DL, Derraik JGB, Robinson E, Cutfield WS, Hofman PL (2011) Preterm birth and the endocrine regulation of growth in childhood and adolescence. Clin Endocrinol 75: 661–665. 10.1111/j.1365-2265.2011.04116.x 21609348

[13] Niklasson A, Ericson A, Fryer J, Karlberg J, Lawrence C, Karlberg P (1991) An update of the Swedish reference standards for weight, length and head circumference at birth for given gestational age (1977–1981). Acta Paediatr 80: 756–762. 1957592 10.1111/j.1651-2227.1991.tb11945.x

[14] Tanner JM, Whitehouse RH (1976) Clinical longitudinal standards for height, weight, height velocity, weight velocity, and stages of puberty. Arch Dis Child 51: 170–179. 952550 10.1136/adc.51.3.170 PMC1545912

[15] Tanner J, Whitehouse R, Marshall W, Carter B (1975) Prediction of adult height from height, bone age, and occurrence of menarche, at ages 4 to 16 with allowance for midparent height. Arch Dis Child 50: 14–26. 164838 10.1136/adc.50.1.14 PMC1544488

[16] Pandolfi C, Zugaro A, Lattanzio F, Necozione S, Barbonetti A, Colangeli MS, et al (2008) Low birth weight and later development of insulin resistance and biochemical/clinical features of polycystic ovary syndrome. Metabolism 57: 999–1004. 10.1016/j.metabol.2008.02.018 18555843

[17] Curhan GC, Willett WC, Rimm EB, Spiegelman D, Ascherio AL, Stampfer MJ (1996) Birth weight and adult hypertension, diabetes mellitus, and obesity in US men. Circulation 94: 3246–3250. 8989136 10.1161/01.cir.94.12.3246

[18] Clausen JO, Borch-Johnsen K, Pedersen O (1997) Relation between birth weight and the insulin sensitivity index in a population sample of 331 young, healthy Caucasians. Am J Epidemiol 146: 23–31. 9215220 10.1093/oxfordjournals.aje.a009188

[19] Doyle LW, Faber B, Callanan C, Morley R (2003) Blood pressure in late adolescence and very low birth weight. Pediatrics 111: 252–257. 12563047 10.1542/peds.111.2.252

[20] Curhan GC, Chertow GM, Willett WC, Spiegelman D, Colditz GA, Manson JE, et al (1996) Birth weight and adult hypertension and obesity in women. Circulation 94: 1310–1315. 8822985 10.1161/01.cir.94.6.1310

[21] Doyle LW, Faber B, Callanan C, Ford GW, Davis NM (2004) Extremely low birth weight and body size in early adulthood. Arch Dis Child 89: 347–350. 15033844 10.1136/adc.2002.025924 PMC1719869

[22] Elliman A, Bryan E, Walker J, Harvey D (1992) The growth of low-birth-weight children. Acta Paediatr 81: 311–314. 1606390 10.1111/j.1651-2227.1992.tb12232.x

[23] Hack M, Schluchter M, Cartar L, Rahman M, Cuttler L, Borawski E (2003) Growth of very low birth weight infants to age 20 years. Pediatrics 112: e30–38. 12837903 10.1542/peds.112.1.e30

[24] Barker DJP (1994) Mothers, Babies and Disease in Later Life. London: BMJ Publishing Group.

[25] Mathai S, Derraik JGB, Cutfield WS, Dalziel SR, Harding JE, Biggs J, et al (2013) Increased adiposity in adults born preterm and their children. PLoS ONE 8: e81840 10.1371/journal.pone.0081840 24278462 PMC3835734

[26] Tinnion R, Gillone J, Cheetham T, Embleton N (2014) Preterm birth and subsequent insulin sensitivity: a systematic review. Arch Dis Child 99: 362–368. 10.1136/archdischild-2013-304615 24363362

[27] Mathai S, Cutfield WS, Derraik JGB, Dalziel SR, Harding JE, Robinson E, et al (2012) Insulin sensitivity and β-cell function in adults born preterm and their children. Diabetes 61: 2479–2483. 22596051 10.2337/db11-1672 PMC3447901

[28] Hofman PL, Regan F, Jackson WE, Jefferies C, Knight DB, Robinson EM, et al (2004) Premature birth and later insulin resistance. N Engl J Med 351: 2179–2186. 15548778 10.1056/NEJMoa042275

[29] Moran A, Jacobs DR Jr, Steinberger J, Cohen P, Hong C-P, Prineas R, et al (2002) Association between the insulin resistance of puberty and the insulin-like growth factor-I/growth hormone axis. J Clin Endocrinol Metab 87: 4817–4820. 12364479 10.1210/jc.2002-020517

[30] Loomba-Albrecht LA, Styne DM (2009) Effect of puberty on body composition. Curr Opin Endocrinol Diabetes Obes 16: 10–15. 19115520 10.1097/med.0b013e328320d54c

